# Beyond the Flare: A Case of Systemic Lupus Erythematosus Complicated by Hemophagocytic Lymphohistiocytosis

**DOI:** 10.7759/cureus.86998

**Published:** 2025-06-29

**Authors:** Juveriya Yasmeen, Dinara Salimova, Nita Lohala, Tatevik Aloyan, Bekure B Siraw

**Affiliations:** 1 Internal Medicine, Ascension Saint Joseph Hospital, Chicago, USA

**Keywords:** autoimmune encephalopathy, hemophagocytic lymphohistiocytosis, systemic autoimmune disease, systemic inflammatory response syndrome (sirs), systemic lupus erythromatosus

## Abstract

Hemophagocytic lymphohistiocytosis (HLH) is a rare, potentially fatal hyperinflammatory syndrome marked by excessive immune activation, cytokine storm, multiorgan failure, and high mortality. Systemic lupus erythematosus (SLE) is considered to be one of the triggers. SLE-associated HLH is especially challenging to diagnose and manage, as it can mimic lupus flares. We describe a 30‑year‑old woman with SLE who developed secondary HLH. Initially thought to have an SLE flare, she rapidly deteriorated with multiorgan dysfunction. The diagnosis of HLH was confirmed using the HLH-2004 criteria and a significantly elevated H-score. Despite intensive treatment with corticosteroids and immunosuppressive agents, the patient ultimately died from HLH-related complications. This case highlights the critical need for early recognition and prompt differentiation of HLH from lupus flares in SLE patients. Timely, targeted therapy is essential to improve outcomes in this high-risk group.

## Introduction

Hemophagocytic lymphohistiocytosis (HLH) is a rare, life-threatening syndrome characterized by excessive immune activation, leading to cytokine release syndrome, hyperinflammation, multiorgan failure, and high mortality rates [1]. First described in 1939 by pediatricians Scott and Robb-Smith, it has been named histiocytic medullary reticulosis or macrophage activation syndrome (MAS), until 1991, when the Histiocyte Society proposed the name HLH [1,2].

HLH is broadly classified into primary (familial) and secondary (acquired) forms [3]. Primary HLH is caused by biallelic mutations in genes critical to cytotoxic lymphocyte function, typically presenting in infancy but not limited to early childhood [3,4]. Secondary HLH arises in response to external triggers such as infections (notably Epstein-Barr virus), malignancies, and autoimmune diseases, with MAS being the term used in the rheumatologic context [3,4].

In critically ill adults, infections constitute the most frequent trigger, followed by malignancies and autoimmune diseases [1]. Notably, among autoimmune-associated HLH cases, systemic lupus erythematosus (SLE) is the most commonly implicated disorder. SLE-associated HLH is particularly challenging due to its overlap with lupus flares and systemic inflammatory conditions like sepsis, often leading to underdiagnosis and delayed treatment [5].

While primary HLH is driven by genetic defects, secondary HLH results from immune dysregulation due to underlying conditions, with both forms characterized by uncontrolled immune activation and cytokine storm [3]. The pathophysiology of acquired HLH is multifactorial, involving a complex interplay of genetic susceptibility and external triggers such as infections, malignancies, or autoimmune disorders [3,4]. Although some patients with acquired HLH carry monoallelic mutations in genes implicated in familial HLH, their pathogenic significance remains uncertain. A hallmark of HLH is sustained CD8+ T-cell activation and macrophage-driven hyperinflammation, leading to excessive cytokine production. This dysregulated immune response may be driven by toll-like receptor activation or inflammasome mutations, resulting in a cytokine storm and the clinical manifestations of HLH [3,4]. This case report presents a 30-year-old female patient with SLE who developed secondary HLH, highlighting the diagnostic complexities, therapeutic challenges, and critical management considerations in this rare but devastating complication.

## Case presentation

A 30-year-old female patient with a known history of SLE, characterized with positive anti-Smith, chromatin, ribonucleoprotein (RNP), Sjogren’s syndrome-related antigen A (SSA), and scleroderma (Scl-70) antibodies, presented to the emergency department with a two-day history of severe bilateral knee arthralgia (rated 8/10), low-grade fever, chest tightness, exertional dyspnea, and a nonproductive cough. She denied associated joint swelling, mucocutaneous ulcerations, alopecia, dysuria, paresthesias, or focal motor deficits. The constellation of symptoms closely resembled her prior SLE flares. Additional complaints included headache exacerbated by head movements and photophobia.

On initial evaluation, the patient was febrile (103°F), tachycardic (heart rate of 120 bpm), and hypotensive (blood pressure of 87/46 mmHg). Laboratory investigations (Table 1) were notable for hypokalemia, hypomagnesemia, mixed-pattern transaminitis, and elevated inflammatory markers (erythrocyte sedimentation rate (ESR) and C-reactive protein (CRP)). Urinalysis was unremarkable. Infectious workup, including SARS-CoV-2, influenza, respiratory syncytial virus (RSV), and methicillin-resistant *Staphylococcus aureus *(MRSA) polymerase chain reaction (PCR), was negative. Chest radiograph was nonrevealing, and computed tomography angiography (CTA) of the chest excluded pulmonary embolism. Electrocardiogram demonstrated sinus tachycardia with nonspecific T-wave changes. The patient was initiated on prednisone 30 mg and hydroxychloroquine 200 mg daily.

**Table 1:** Laboratory values on day 1 of admission WBC: white blood cell; RBC: red blood cell; MCV: mean corpuscular volume; MCH: mean corpuscular hemoglobin; MCHC: mean corpuscular hemoglobin concentration; RDW: red blood cell distribution width; MPV: mean platelet volume; BUN: blood urea nitrogen; ALT: alanine transaminase; AST: aspartate transaminase; ALP: alkaline phosphatase; ESR: erythrocyte sedimentation rate; CRP: C-reactive protein

Component	Value	Normal range
WBC	4.0 k/mm³	4.0-11.0
Platelets	180 k/mm³	150-450
RBC	4.29 m/mm³	3.63-5.04
Hemoglobin	12.4 g/dL	12.0-15.3
Hematocrit	37.2%	34.7-45.1
MCV	86.7 fL	80.0-100.0
MCH	28.9 pg	26.0-34.0
MCHC	33.3%	32.5-35.8
RDW	14.1%	11.9-15.9
MPV	8.4 fL	6.8-10.2
Glucose	117 mg/dL	70-99
BUN	16 mg/dL	7-25
Creatinine	1 mg/dL	0.6-1.2
Sodium	136 mmol/L	133-144
Potassium	3.3 mmol/L	3.5-5.2
Magnesium	1.4 mg/dL	1.6-2.6
ALT	215 IU/L	7-52
AST	293 IU/L	13-39
ALP	95 IU/L	34-104
Total bilirubin	0.7 mg/dL	0.3-1.0
Total protein	7.7 g/dL	6.4-8.9
Albumin	3.7 g/dL	3.5-5.7
Lactic acid	1.7 mmol/L	0.7-2.0
ESR	31 mm/hr	0-30
CRP	7.7 mg/dL	<1.0
Lipase	11 IU/L	11-82
High sensitivity troponin	15 pg/mL	0-12

Given persistent fever, hypotension, and tachycardia, a lumbar puncture was performed. Cerebrospinal fluid (CSF) analysis showed elevated protein of 117 mg/dL, neutrophilic pleocytosis (90% neutrophil, 8% lymphocytes, 1% monocytes, 1% eosinophils), and normal glucose level of 68 mg/dL (Table 2). Cultures yielded no microbial growth and a negative extended viral/bacterial meningitis/encephalitis panel. Rheumatology recommended escalation of corticosteroid therapy to intravenous methylprednisolone 100 mg every eight hours, in light of a presumed lupus flare. Gastroenterology was consulted for transaminitis, and a right upper quadrant ultrasound revealed mild hepatomegaly. Viral hepatitis panel, acetaminophen levels, and autoimmune liver serologies (anti-smooth muscle antibodies (AMA) were within normal limits. The Model for End-Stage Liver Disease (MELD)-sodium (Na) score was calculated at 17.

**Table 2:** Cerebrospinal fluid study results CSF: cerebrospinal fluid; TNC: total nucleated cell count; RBC: red blood cell

CSF component	Value	Normal range
Appearance	Clear	Clear
Color	Colorless	Colorless
Pressure	<200 mm Hg	<200
Glucose	68 mg/dL	40-70
Protein	117 mg/dL	15-45
TNC	19 cells/µL	0-5
Neutrophils	90%	0
Lymphocytes	8	66-99
Monocytes	1%	3-37
Eosinophils	1%	0
RBC	6/cu mm	0-10

On hospital day 2, white blood cell count increased from 4,000 to 11,000/µL. Blood cultures remained sterile. Autoantibody profiling confirmed anti-nuclear antibody (ANA) positivity with elevated SSA (>8), anti-chromatin IgG (7.5), anti-Smith (1.6), RNP (>8), and Scl-70 (1.3). Complement levels (C3, C4) were within the normal range. Monospot (for EBV) and CMV IgM were negative. High-sensitivity troponin levels rose from 15 ng/L on presentation to a peak of 15,000 ng/L before trending down to 10,000 ng/L. Electrocardiogram (EKG) findings of subtle ST elevations and PR depression prompted echocardiographic evaluation, which revealed preserved left ventricular systolic function (ejection fraction of 61%) and mild-to-moderate mitral regurgitation. Lupus myocarditis and pericarditis were suspected, prompting escalation to pulse-dose methylprednisolone (1 g IV daily).

Nephrology was involved for acute kidney injury and hyponatremia. Renal ultrasound was unremarkable. On day 5, following three days of pulse corticosteroids, prednisone was tapered to 60 mg daily. Colchicine was initiated for presumed lupus myopericarditis. Hydroxychloroquine was increased to 200 mg twice daily. On day 6, the patient developed progressive encephalopathy. Laboratory evaluation revealed elevated serum ammonia (71 µmol/L), raising concern for hepatic encephalopathy versus lupus cerebritis. Lactulose and thiamine were initiated. Prednisone was switched to oral prednisolone (due to acute liver injury).

On hospital day 7, persistent transaminitis warranted an image-guided liver biopsy. Post-procedure, the patient acutely decompensated with tachycardia, tachypnea, hypotension, and a hemoglobin drop to 6.8 g/dL. Serum lactate was elevated to 4.7 mmol/L. Point-of-care ultrasound demonstrated intra-abdominal free fluid. Abdominal CTA revealed moderate hemoperitoneum without evidence of active bleeding. The patient was transfused with 2 units of packed red blood cells and 1 unit of fresh frozen plasma (international normalized ratio (INR) of 1.7). Given hemodynamic stability and absence of active hemorrhage, conservative management was pursued.

On hospital day 8, the patient developed severe rhabdomyolysis (creatine kinase, >20000 U/L) for which colchicine was discontinued, and aggressive intravenous hydration was initiated. Laboratory data (Table 3) revealed markedly elevated serum ferritin (>15000 ng/mL), hypertriglyceridemia (646 mg/dL), and persistent high-grade fever. Further evaluation revealed elevated serum soluble IL-2 receptor (6526 pg/mL), hypofibrinogenemia (77 mg/dL), and markedly reduced NK cell activity (total count 10/µL), fulfilling five out of eight diagnostic criteria for HLH per the HLH-2004 guidelines. HScore, a diagnostic tool for reactive hemophagocytic syndrome that is described in detail below, was calculated at 257, significantly exceeding the diagnostic threshold of 169. Bone marrow biopsy was deferred due to the patient's critical status. Azathioprine 50 mg daily was initiated, while considerations were being given to the Etoposide pel HLH-2004 protocol.

**Table 3:** Laboratory values on day 8 of admission

Component	Values	Normal range
Creatine kinase	>20000 U/L	18-225
Ferritin	15000 ng/mL	16-288
Fibrinogen	77 mg/dL	175-425
Triglycerides	646 mg/dL	<150
Soluble IL-2 receptor	6526 pg/mL	532-1891

By hospital day 9, the patient exhibited worsening hepatic encephalopathy, cytopenias, escalating rhabdomyolysis, and multiorgan dysfunction. She experienced a pulseless electrical activity (PEA) cardiac arrest, achieving return of spontaneous circulation after 10 minutes of cardiopulmonary resuscitation. Post-arrest echocardiogram revealed a severely reduced ejection fraction (15%), consistent with stress cardiomyopathy or post-arrest myocardial stunning. The patient developed progressive multiorgan failure, including cardiogenic shock necessitating multiple vasopressor support, severe anion gap metabolic acidosis, acute kidney injury requiring continuous venovenous hemofiltration (CVVH), acute liver failure, and refractory hypoxemic respiratory failure while on mechanical ventilation. Due to the ongoing hemorrhagic risk and multiorgan dysfunction, the patient was deemed unsuitable for extracorporeal membrane oxygenation (ECMO). Following discussion with the family, goals of care were readdressed, and her code status was changed to do-not-resuscitate (DNR). The patient expired the following day.

## Discussion

This case of a 30-year-old female patient with SLE who developed secondary HLH illustrates the devastating potential of this rare syndrome and the diagnostic and therapeutic challenges it poses. Importantly, HLH shares overlapping immunopathological mechanisms with conditions such as sepsis, systemic inflammatory response syndrome (SIRS), and MAS, often rendering them clinically indistinguishable [6,7]. Our patient initially presented with signs suggestive of an SLE flare, accompanied by laboratory evidence of systemic inflammation. Her course rapidly deteriorated with features of multiorgan involvement, including myopericarditis, acute kidney injury, rhabdomyolysis, and neuropsychiatric symptoms suggestive of cerebritis or hepatic encephalopathy. This is consistent with secondary HLH in the background of an underlying SLE flare.

The diagnosis of HLH is challenging due to nonspecific clinical features and significant overlap with other conditions. At present, there are no unique clinical or laboratory features that have enough sensitivity and specificity to allow an unambiguous HLH diagnosis. The presence of hemophagocytosis features on the bone marrow aspiration or other tissue biopsy was initially considered a gold standard for the diagnosis of HLH. However, hemophagocytic features were also described in patients with other conditions who did not have HLH, such as sepsis, and there are no widely accepted cytologic features specific enough for the diagnosis of HLH. Notably, functional and genetic testing are not routinely recommended in adult HLH cases, as pathogenic mutations are rarely identified in this population [8].

The current strategy for the diagnosis in adults is mainly based on the revised HLH-2004 criteria derived from HLH-94 criteria from the Histiocyte Society. Patients traditionally should have a constellation of at least five out of eight clinical and laboratory criteria (Table 4) [7]. Our patient met five out of eight HLH-2004 criteria. 

**Table 4:** Revised HLH-2004 criteria HLH: hemophagocytic lymphohistiocytosis; NK cell: natural killer cell

Diagnostic criteria for HLH
Fever
Splenomegaly
Cytopenias (affecting ≥2 of 3 lineages in the peripheral blood): hemoglobin <90 g/L (hemoglobin <100 g/L in infants <4 weeks); platelets <100 × 10^9^/L; neutrophils <1.0 × 10^9^/L
Hypertriglyceridemia and/or hypofibrinogenemia: fasting triglycerides ≥3.0 mmol/L (i.e., ≥265 mg/dL); fibrinogen ≤1.5 g/L
Hemophagocytosis in bone marrow or spleen or lymph nodes. No evidence of malignancy
Low or no NK cell activity (according to local laboratory reference)
Ferritin ≥500 μg/L
sCD25 (i.e., soluble IL-2 receptor) ≥2400 U/mL

In 2014, Fardet et al. proposed the HScore (Table 5), a diagnostic tool for reactive hemophagocytic syndrome, based on a multicenter retrospective cohort of 312 patients, with an optimal cutoff of 169 (sensitivity, 93%; specificity, 86%) [8]. Our patient’s HScore of 257, driven by fever, cytopenias, hyperferritinemia, hypertriglyceridemia, hepatomegaly, transaminitis, and underlying SLE, robustly supported the diagnosis.

**Table 5:** HScore, a diagnostic tool for reactive hemophagocytic syndrome

Parameter	No. of points (criteria for scoring)
Known underlying immunosuppression	0 (no) or 18 (yes)
Temperature (°C)	0 (<38.4), 33 (38.4–39.4), or 49 (>39.4)
Organomegaly	0 (no), 23 (hepatomegaly or splenomegaly), or 38 (hepatomegaly and splenomegaly)
No. of cytopenias	0 (1 lineage), 24 (2 lineages), or 34 (3 lineages)
Ferritin (μg/L)	0 (<2000), 35 (2000-6000), or 50 (>6000)
Triglyceride (mmol/L)	0 (<1.5), 44 (1.5-4), or 64 (>4)
Fibrinogen (g/L)	0 (>2.5) or 30 (≤2.5)
Aspartate aminotransferase (U/L)	0 (<30) or 19 (≥30)
Hemophagocytosis in bone marrow aspirate	0 (no) or 35 (yes)

The significant clinical overlap between HLH, severe sepsis, and SLE flares posed a major diagnostic challenge. Although the patient’s presentation resembled sepsis, an extensive infectious workup, including blood cultures, viral panels, and lumbar puncture, was negative. The detection of SLE-specific autoantibodies (ANA, SSA, Smith, RNP) and the lack of an infectious etiology raised suspicion for MAS, a subtype of secondary HLH associated with autoimmune disease. MAS in SLE is thought to arise from both genetic predispositions (e.g., IRF5 or NLRC4 mutations) and immune triggers, which together amplify innate immune responses and cytokine release (including IL-1, IL-6, IL-18) [8,9]. Ali et al. [10], Alharbi et al. [11], and Egües Dubuc et al. [12], along with other researchers, identified SLE-associated HLH in their patients based on the criteria outlined above.

Therapeutic management of SLE-associated HLH requires a dual approach: addressing the underlying trigger (e.g., autoimmune flare) and suppressing the deleterious immune response. In this case, initial treatment targeted the presumed SLE flare with high-dose corticosteroids and hydroxychloroquine, consistent with guidelines for MAS [3,9]. Colchicine was added for lupus-induced myopericarditis, and azathioprine was initiated late in the course as HLH suspicion grew.

The standardized HLH-94 protocol has improved prognosis from a one-year survival rate of less than 5% to a five-year survival rate of 21% [13,14]. HLH-94 was initially developed for the pediatric population. It includes an eight-week course of etoposide and dexamethasone. HLH-94 protocol also includes hematopoietic stem cell transplantation (HSCT) and an intrathecal methotrexate administration for progressive neurologic symptoms [13,14]. HLH-2004 protocol represents modified HLH-94 and includes a similar course of etoposide with dexamethasone, with the addition of cyclosporine A.

The standardized HLH-94 and HLH-2004 protocols were not initiated in our patient. This was likely due to delayed recognition of HLH, as the initial focus on an SLE flare and sepsis workup postponed specific HLH-directed therapy, compounded by the patient’s rapid deterioration and procedural complications (e.g., post-biopsy hemoperitoneum), which limited the feasibility of cytotoxic agents.

Our patient’s rapid deterioration following an image-guided liver biopsy highlights the risks of invasive procedures in critically ill patients with coagulopathy. The fatal outcome highlights the high mortality of SLE-associated HLH (9.9% in hospitalized cases per Noboa et al.) [14].

## Conclusions

This case highlights several critical lessons for managing SLE-associated HLH. First, clinicians must maintain a high index of suspicion in SLE patients with persistent fever, cytopenias, and multiorgan dysfunction, using tools like the HScore and HLH-2004 criteria. Second, early multidisciplinary consultation (rheumatology, hematology, critical care) and prompt immunosuppression are essential to improve outcomes. Third, invasive procedures should be approached cautiously to avoid catastrophic complications. Future research into targeted therapies, such as IL-1 or IL-6 inhibitors, and standardized protocols for adult HLH is warranted to address diagnostic delays and therapeutic limitations, ultimately reducing the devastating impact of this condition.
